# Patterns of peritoneal dialysis catheter practices and technique failure in peritoneal dialysis: A nationwide cohort study

**DOI:** 10.1371/journal.pone.0218677

**Published:** 2019-06-20

**Authors:** Antoine Lanot, Clemence Bechade, Christian Verger, Emmanuel Fabre, Isabelle Vernier, Thierry Lobbedez

**Affiliations:** 1 Normandie Univ, UNICAEN, CHU de Caen Normandie, Néphrologie, CAEN, France; 2 Normandie université, Unicaen, UFR de médecine, Caen, France; 3 RDPLF, Pontoise, France; 4 Néphrologie, polyclinique le Languedoc, Narbonne, France; University of Utah School of Medicine, UNITED STATES

## Abstract

**Introduction:**

Our objective was to assess whether clusters of centers with similar peritoneal dialysis (PD) catheter related practices were associated with differences in the risk of technique failure.

**Methods:**

Patients on incident PD in French centers contributing to the French Language PD Registry from 2012 to 2016 were included in a retrospective analysis of prospectively collected data. Centers with similar catheter cares practices were gathered in clusters in a hierarchical analysis. Clusters of centers associated with technique failure were evaluated using Cox and Fine and Gray models. A mixed effect Cox model was used to assess the influence of a center effect, as explained by the clusters.

**Results:**

Data from 2727 catheters placed in 64 centers in France were analyzed. Five clusters of centers were identified. After adjustment for patient-level characteristics, the fourth cluster was associated with a lower risk of technique failure (cause specific-HR 0.70, 95%CI 0.54–0.90. The variance of the center effect decreased by 5% after adjusting for patient characteristics and by 26% after adjusting for patient characteristics and clusters of centers in the mixed effect Cox model. Favorable outcomes were observed in clusters with a greater proportion of community hospitals, where catheters were placed via open surgery, first dressing done 6 to 15 days after catheter placement, and local prophylactic antibiotics was applied on exit-site.

**Conclusion:**

Several patterns of PD catheter related practices have been identified in France, associated with differences in the risk of technique failure. Combinations of favorable practices are suggested in this study.

## Introduction

One of the main challenges of peritoneal dialysis (PD) is to ensure the sustainability of the technique. Peritonitis is a leading cause of technique failure [1], [2], [3]. Individual risks factors for technique failure and peritonitis have been identified [4], [5], [6]. It has been demonstrated that a center effect explained by center characteristics influences the risks of peritonitis and technique failure [7], [8], [9]. This is a matter of concern since center characteristics are modifiable. PD practices are heterogeneous and variations observed between PD units contribute to the risk of peritonitis [10], [11], [12]. Consequently quality improvement programs at the center level are necessary [7], [13]. Identifying groups of centers with higher risks of peritonitis or technique failure is of importance if one wants to prioritize the action at the nationwide level. Furthermore practices may be interrelated and influence the patient outcomes.

We identified clusters of French PD centers that were grouped according to the proximity of their PD catheter related practices [10]. We hypothesize that these patterns of practices identified by a hierarchical analysis, may affect the patient outcome on PD. The objective of this study was to determine whether those clusters of centers with similar practices are associated with the risks of technique failure, peritonitis, and technique failure due to peritonitis.

## Materials and methods

### Study population

This was a retrospective study using data from the "catheter" section of the French Language Peritoneal Dialysis Registry (RDPLF) [14]. Eighty-seven centers provide data for the optional catheter section. Centers where fewer than 5 catheters were registered, and centers located in French overseas territories were excluded. Patients older than 18 years starting PD in France between 1 January 2012 and 31 December 2016 were included in the study. The end of study period was 3 February 2017.

### Definition of variables

#### Individual characteristics (level 1 covariates)

Age, sex, weight, obesity (defined as a body mass index greater than 30 kg/m^2^), malnutrition (defined as a composite of BMI < 18.5 kg/m2 or < 21 kg/m2 for subjects older than 70 years old, and hypoalbuminemia, according to the pathology), causal nephropathy, existence of diabetes and its type of treatment, and modified Charlson comorbidity index, which is the Charlson comorbidity index after excluding the age subscore, were extracted from the registry.

#### Center practices and clusters of centers (level 2 covariates)

Type of PD catheter, surgery technique, administration of antibiotic prophylaxis prior to catheter insertion, specialized surgeon for catheter placement, screening for nasal carriage of *S*. *aureus*, use of prophylactic nasal antistaphylococcal cream, use of local antistaphylococcal cream or ointment on the catheter emerging site, delay after catheter insertion for first dressing, type of antiseptic for dressing refection, PD modality 3 months after dialysis initiation (automated peritoneal dialysis [APD] or continuous ambulatory peritoneal dialysis [CAPD]), assistance for PD (self-PD, family assistance, nurse assistance), type of administrative structure, and center size (number of catheters registered) were obtained from the database.

For each center, a practice was defined as being the center standard practice during the follow-up period whenever it was applied in 75% or more of the listed cases, from the individual data available in the registry.

A hierarchical clustering analysis using Ward’s method was applied to determine five clusters of centers with similar practices [15]. Each practice is thought of as a specific dimension of a virtual space, and the different modalities of the practice constitute the gradations along this dimension. A space of practices is therefore built, in which each center has a location according to its practices (S1 Fig). A Euclidian distance is defined in this virtual space and computed between the centers. Then, a dendrogram is built, joining centers with nodes at height proportional to the proximity of their practices. This allows the constitution of clusters of centers with similar practices. The number of clusters is determined graphically according to the semi-partial R-squared graph, to optimize the variability between clusters while minimizing complexity.

### Study outcomes

The primary outcome was PD technique failure, defined as a transfer to hemodialysis for longer than 2 months. Two events of interest were considered as secondary outcomes: first peritonitis episode for a given PD catheter, and technique failure due to peritonitis. A supplemental analysis was performed computing the risks for early or late technique failure (occurring respectively earlier or later than 3 months after the starting of PD). A composite outcome of technique failure and mortality was assessed to identify covariates potentially decreasing the risk of technique failure while increasing mortality.

### Statistical analysis

Categorical variables were described by their frequencies and percentages and continuous variables by their median and interquartile ranges. Differences of the distribution of practices among the clusters were evaluated using chi-squared tests. We examined for collinearity by assessing the generalized variance inflation factor.

### Survival analysis

#### Cox modeling

Kaplan-Meier survival curves were drawn, and log-rank tests were performed to determine whether the outcomes were different between the five clusters. Cox models were used to explore the association between the covariates and the events of interest: technique failure, first peritonitis episode, early and late technique failure. Considering the event technique failure due to peritonitis, other causes of technique failure would have been censored in a classical Cox model, overestimating the HR, so this event was not considered in the Cox model. Cause-specific HR with 95% confidence intervals (CI) were estimated. Regression splines were used to explore the effect of continuous variables and to choose their management in the models. Proportional hazard assumption was tested by visual inspection of Schoenfeld residual plots. Covariates were entered in the multivariate analysis when p < 0.20 in the bivariate analysis, the cluster covariate was entered a priori since it was the covariate of interest.

#### Fine and Gray modeling

To avoid overestimating hazard rate, a competing risk analysis was performed, accounting for the risk of death, renal transplantation and other causes of technique failure [16]. The Fine and Gray model allows the estimation of the subdistribution HR, which is defined as the hazard of the event of interest in the presence of a competing event. The Fine and Gray modeling was used to explore the association between the covariates and all the outcomes of interest.

#### Hierarchical analysis

To assess the relevance of the approach with clusters of practices, we used a hierarchical analysis. A mixed-effect Cox model was used to estimate the influence of the clusters on the center effect for the two events: technique failure, and first peritonitis episode. An empty Cox model (model 0) with center as random effect was fitted to estimate the random effect. Individual characteristics (level 1 covariates) were included in the model (model 1) to investigate whether the heterogeneity between centers was explained by the patient composition of the center. Cluster (level 2 covariates) was included in the model (model 2) to determine the magnitude of the clusters on the center effect. Model 1 and model 2 were compared with model 0 by analysis of variance (ANOVA). The proportional change in variance was calculated to estimate the contribution of the covariates in each model. Individual characteristics (level 1 covariates) were entered a priori in the multivariate analysis to improve the assessment of the center effect.

#### Validation analysis

We performed Cox and Fine and Gray analysis for each practice at the patients’ level to assess the isolated effect of practices on technique failure risk. Results were compared with those found in the 4^th^ cluster of centers.

The rate of missing data was < 3%. Given this very low rate, a complete case analysis was performed.

Statistical analyses were performed with R 3.1.1 (R Foundation for Statistical Computing, Vienna, Austria) including the hclust, survival, cmprsk and coxme packages.

The RDPLF provides an informative note to the patients, explaining the use made of their data, their rights to get an access to these data. Patients are informed of their right to oppose to the collection of their data without any consequence on their treatment. Nephrologists in the centers participating to the registry have the responsibility to give this note to any included patient, to obtain the patient’s signature for agreement, and to keep this agreement in the medical records. The RDPLF has the approval of the French National Ethics Committee (*Commission nationale de l’informatique et des libertés*, *agreement number 542 668*) and fulfills the GDPR requests). This study took place within the framework of this authorization. All data analyzed were fully anonymized by the RDPLF, before we accessed them.

This study was reported in accordance with the Strengthening the Reporting of Observational Studies in Epidemiology (STROBE) guidelines [17].

## Results

### Patient characteristics (level 1 covariates)

**Fig 1:**
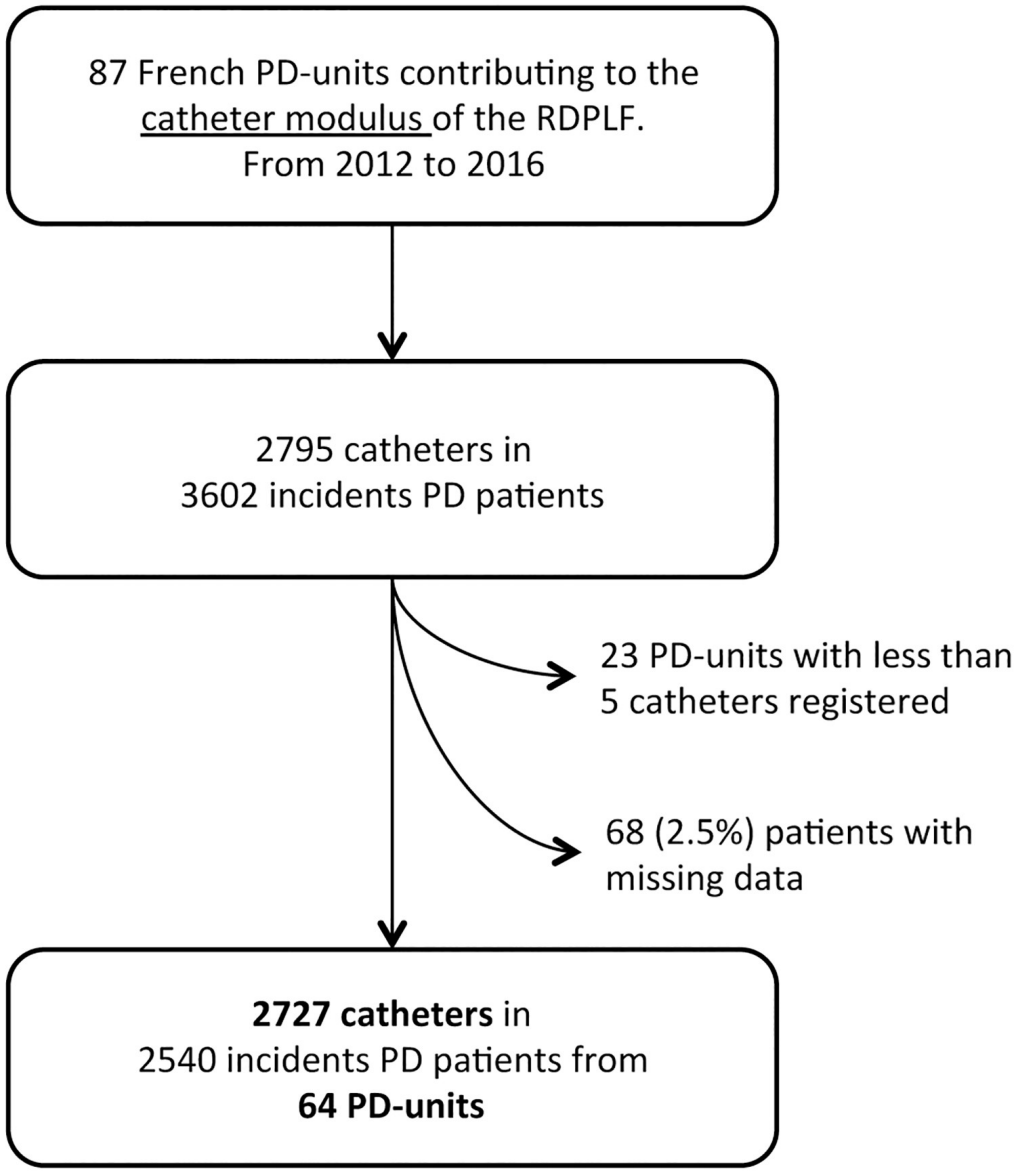
Flowchart.

Data concerning 2727 PD catheters placed in 2540 patients were included from 64 centers (Fig 1). The proportion of men was 1661/2727 (61%), median age was 68 years (first and third quartiles, 54 and 80), and median weight was 70.5 kg (first and third quartiles, 61 and 82). Of the 2727 patients 878 (35%) were diabetic.

### Center characteristics and clusters of practices (level 2 covariates)

**Fig 2:**
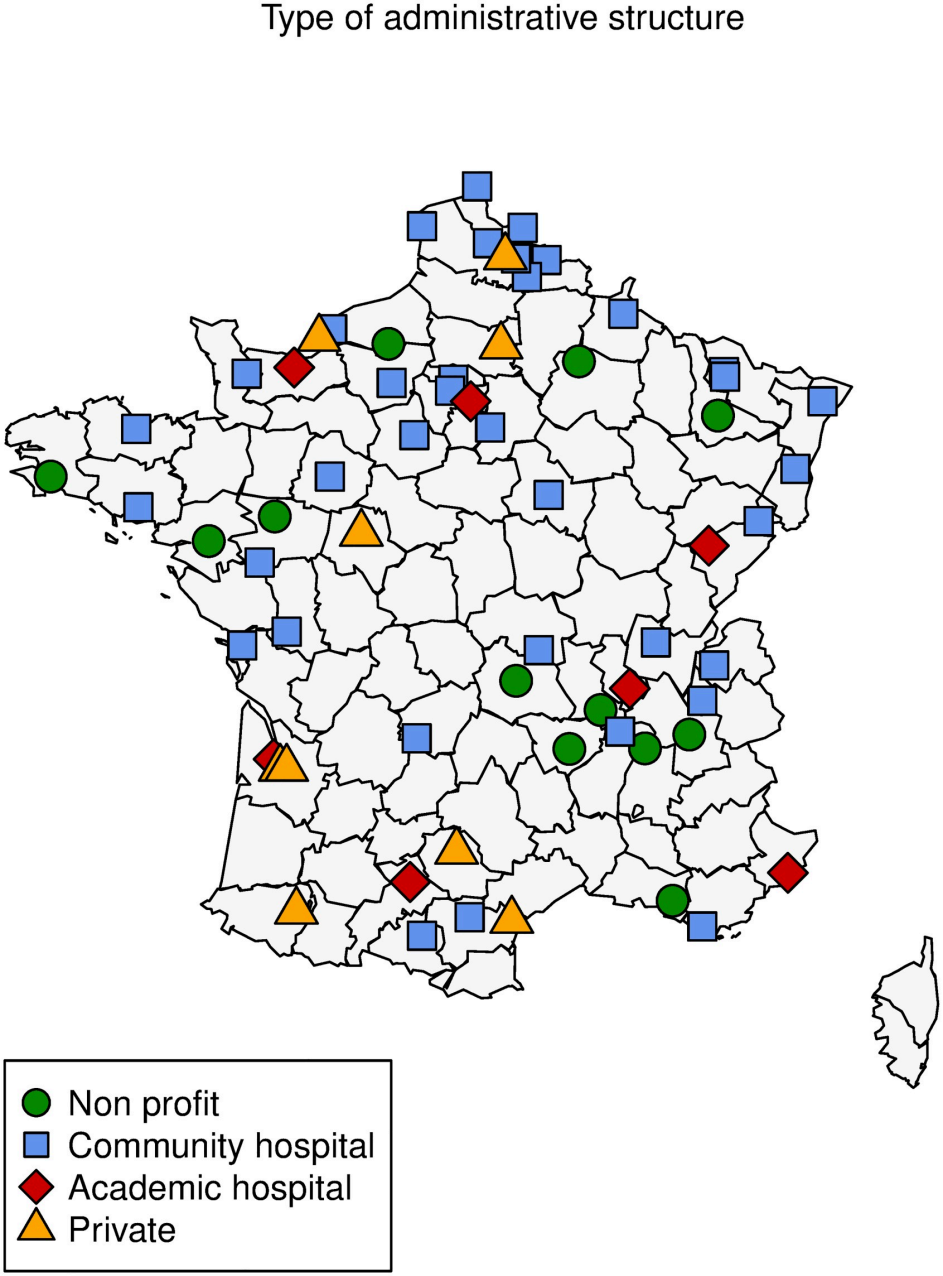
Type of administrative structures.

There were 10 academic hospitals, 33 community hospitals, 12 nonprofit centers, and 9 private centers (Fig 2). The number of catheters placed and listed in each center ranged from 5 to 132 (median = 34, first and third quartiles = 15–61). No collinearity was detected between the practices with generalized variance index factors < 5.

**Fig 3:**
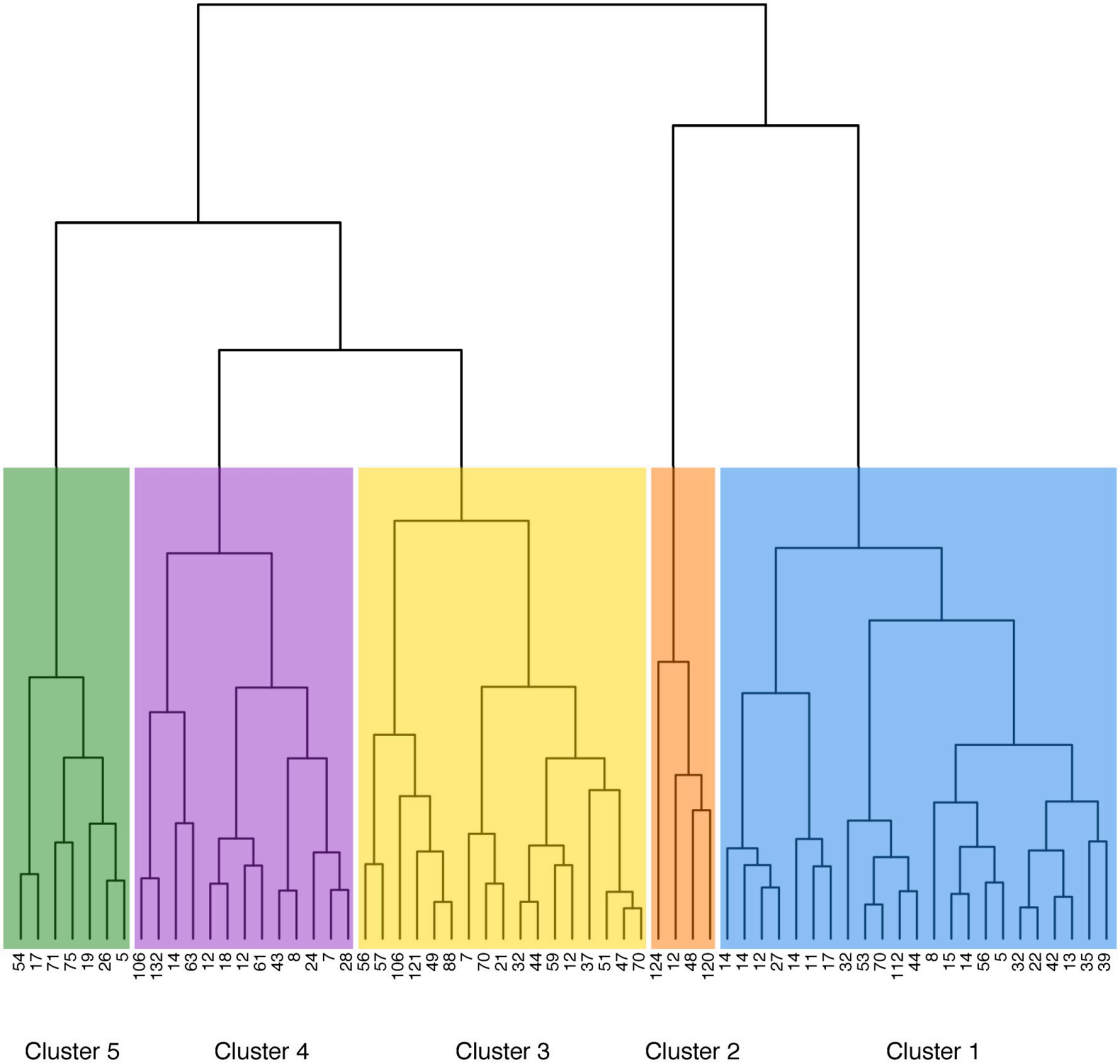
Dendrogram defining the clusters of centers. Each PD-center is represented by a black dot at the bottom of the dendrogram, with the number of catheter registered in it. In the virtual space of practices, distances between each center are computed. Centers are joined by nodes placed at a height that is proportional to the distance between the centers. Five clusters of centers are determined to optimize the variability between clusters while minimizing complexity.

**Fig 4:**
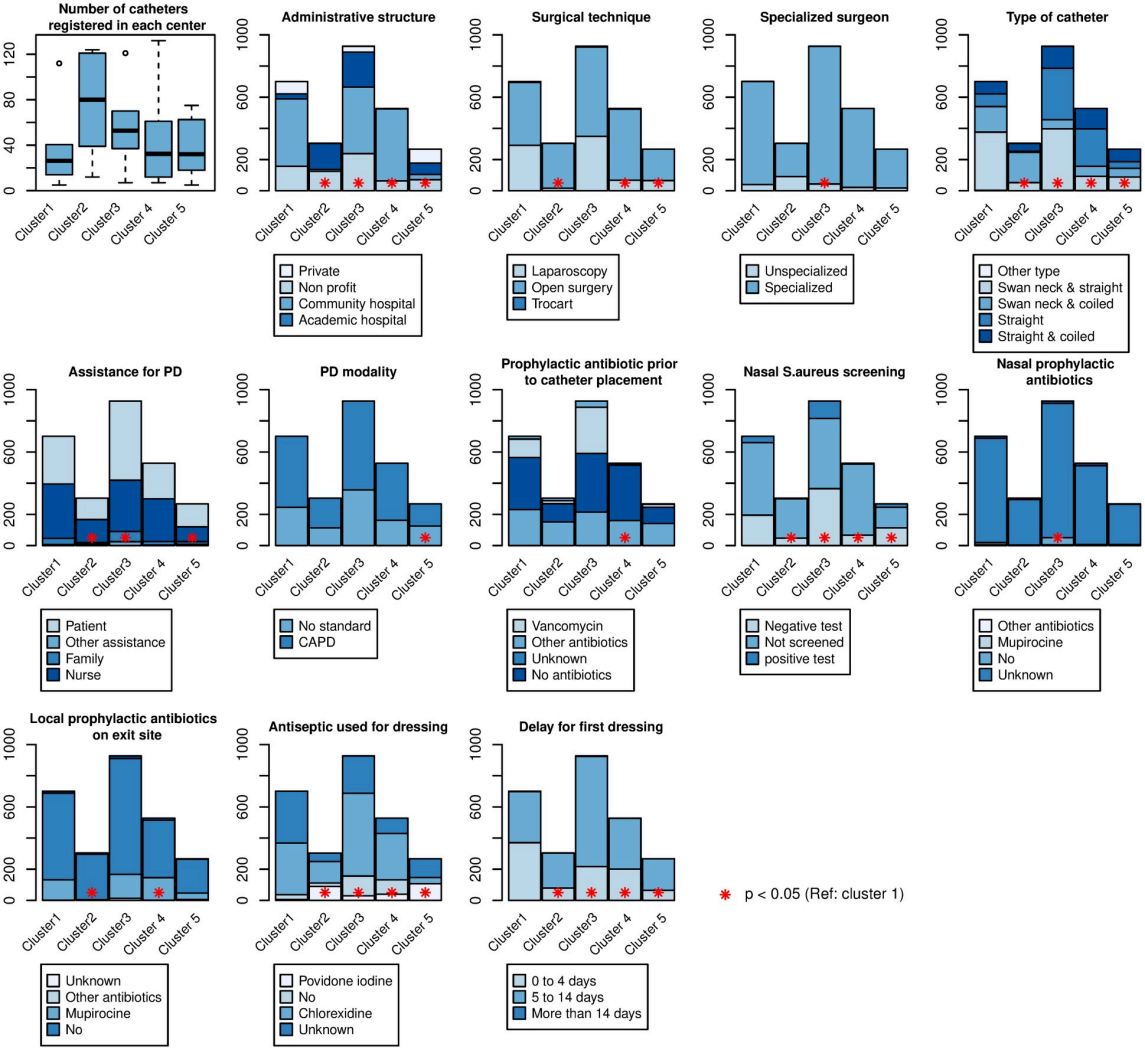
Distribution of practices in the five clusters of centers. The distribution of the modalities used for each practices is represented in the five clusters of centers. Statistically significant differences in the distribution of modalities with cluster 1 (taken as the reference) are marked with a red star.

Five clusters of centers have been identified, according to the similarity of their PD catheter related practices (Fig 3). Table 1 describes the distribution of patient characteristics among the five clusters of centers. Center characteristics and their distribution among the five clusters are given in Table 2 and illustrated in Fig 4.

**Table 1:** Distribution of patient-level characteristics in the five clusters of centers.

		Cluster 1	Cluster 2	Cluster 3	Cluster 4	Cluster 5
		N = 701	N = 304	N = 927	N = 528	N = 267
Patient characteristics						
Sex: male (%)		427 (61)	197 (65)	538 (58)	336 (64)	163 (61)
Weight in kg	Median (IQR)	71 (62–82)	75 (62–86)	70 (60–80)	72 (63–84)	67 (59–77)
Obesity: N (%)		80 (11)	38 (12)	93 (10)	102 (19)	14 (5)
Malnutrition: N (%)		79 (11)	32 (11)	44 (5)	104 (20)	18 (7)
Age at PD initiation	Median (IQR)	71 (58–81)	66 (54–78)	65 (51–79)	73 (59–81)	67 (52–78)
Nephropathy: N (%)	Diabetic	156 (22)	55 (18)	187 (20)	94 (18)	31 (12)(38)
	GN	66 (9)	30 (10)	83 (9)	55 (10)	26 (10)
	Unknown	67 (10)	30 (10)	78 (8)	46 (9)	47 (18)
	TIN	31 (4)	16 (5)	35 (4)	21 (4)	15 (6)
	ADPKD	34 (5)	21 (7)	73 (8)	29 (5)	28 (10)
	Urologic	3 (0)	4 (1)	16 (2)	29 (5)	1 (0)
	Vascular	95 (14)	10 (3)	91 (10)	68 (13)	17 (6)
	Other	249 (36)	138 (45)	364 (39)	208 (39)	102 (38)
Diabetes: N (%)	Oral treatment	37 (5)	23 (8)	39 (4)	23 (4)	11 (5)
	Insulin	194 (30)	98 (29)	264 (29)	161 (31)	66 (21)
	Diet	22 (3)	12 (4)	35 (4)	25 (5)	14 (5)
Modified CCI	Median (IQR)	4 (2–5)	3 (2–5)	3 (2–5)	4 (2–5)	3 (2–5)

N: Number; IQR: Interquartile range; PD: Peritoneal Dialysis; GN: Glomerulonephritis; TIN: Tubulo interstitial nephropathy. ADPKD: Autosomic dominant polycystic kidney disease. IP: Intra peritoneal. SC: Sub cutaneous. CCI: Charlson Comorbidity Index

**Table 2:** Distribution of center-level characteristics in the five clusters of centers.

		Cluster 1	Cluster 2	Cluster 3	Cluster 4	Cluster 5
		N = 701	N = 304	N = 927	N = 528	N = 267
Administrative structure	Non profit	157 (22)	124 (41)	238 (26)	63 (12)	71 (27)
	Community hosp.	432 (62)	12 (4)	427 (46)	462 (88)	33 (12)
	Academic hosp.	32 (5)	168 (55)	222 (24)	3 (1)	73 (27)
	Private	80 (11)	0	38 (4)	0	90 (34)
Catheters registered	Median (IQR)	22 (14–41)	84 (39–121)	51 (37–70)	24 (12–61)	26 (18–63)
Surgical technique for catheter placement	Laparoscopy	292 (42)	16 (5)	349 (38)	67 (13)	65 (24)
	Open surgery	403 (57)	287 (94)	573 (62)	456 (86)	202 (76)
	Trocart	6 (1)	1 (0)	5 (1)	5 (1)	0
Specialized surgeon		662 (94)	213 (70)	884 (95)	507 (96)	250 (94)
Catheter type	Swan neck—straight	374 (53)	52 (17)	394 (43)	91 (17)	85 (32)
	Swan neck—coiled	164 (23)	195 (64)	59 (6)	65 (12)	56 (21)
	Straight—straight	81 (12)	7 (2)	329 (35)	240 (45)	42 (16)
	Straight—coiled	80 (11)	50 (16)	142 (15)	131 (25)	81 (30)
	Other	2 (0)	0	3 (0)	1 (0)	3 (1)
Prophylactic antibiotics prior to catheter placement	Vancomycin	118 (17)	20 (7)	296 (32)	2 (0)	19 (7)
	Other antibiotics	231 (33)	152 (50)	215 (23)	161 (30)	141 (53)
	No antibiotic	334 (48)	116 (38)	376 (41)	355 (67)	104 (39)
	Unknown	18 (3)	16 (5)	40 (4)	10 (2)	3 (1)
Nasal S. aureus screening	Not screened	465 (66)	252 (83)	451 (49)	455 (86)	132 (49)
	Positive test	41 (6)	4 (1)	111 (12)	6 (1)	8 (21)
	Negative test	195 (28)	48 (16)	365 (39)	67 (13)	114 (43)
Nasal prophylactic antibiotics	No	669 (95)	292 (96)	861 (93)	506 (96)	258 (97)
	Mupirocine	14 (2)	3 (1)	46 (5)	5 (1)	5 (2)
	Other antibiotics	5 (1)	0	5 (1)	2 (0)	1 (0)
	Unknown	13 (2)	9 (3)	15 (2)	15 (3)	3 (1)
Local prophylactic antibiotics on exit-site	No	556 (79)	292 (96)	743 (80)	368 (70)	217 (81)
	Mupirocine	130 (19)	3 (1)	154 (17)	144 (27)	41 (15)
	Other antibiotics	2 (0)	1 (0)	13 (1)	2 (0)	6 (2)
	Unknown	13 (2)	8 (3)	17 (2)	14 (3)	3 (1)
Delay for first dressing	0 to 5 days	370 (53)	79 (26)	217 (23)	201 (38)	64 (24)
	6 to 15 days	328 (47)	224 (74)	706 (76)	325 (62)	203 (76)
	After day 16	3 (0)	1 (0)	4 (0)	2 (0)	0
Antiseptic used for dressing	No antiseptic	31 (4)	7 (22)	127 (14)	92 (17)	0
	Povidone iodine	6 (1)	89 (29)	29 (3)	40 (8)	107 (40)
	Chlorexidine	331 (47)	138 (45)	531 (57)	298 (56)	40 (15)
	Unknown	333 (48)	55 (18)	240 (26)	98 (19)	120 (45)
Assistance for PD	Family	40 (6)	9 (3)	65 (7)	22 (4)	17 (6)
	Nurse	349 (50)	147 (48)	329 (35)	274 (52)	95 (36)
	Patient	306 (44)	137 (45)	508 (55)	228 (43)	147 (55)
	Other assistance	6 (1)	11 (4)	25 (3)	4 (1)	8 (3)
PD modality	APD	246 (35)	114 (38)	357 (39)	162 (31)	125 (47)
	CAPD	455 (65)	190 (62)	570 (61)	366 (69)	142 (53)

Hosp: hospital. TAP: Transverse Abdominal Plane. APD: Automated peritoneal dialysis. CAPD: Continuous ambulatory peritoneal dialysis.

### Events of interest

**Fig 5:**
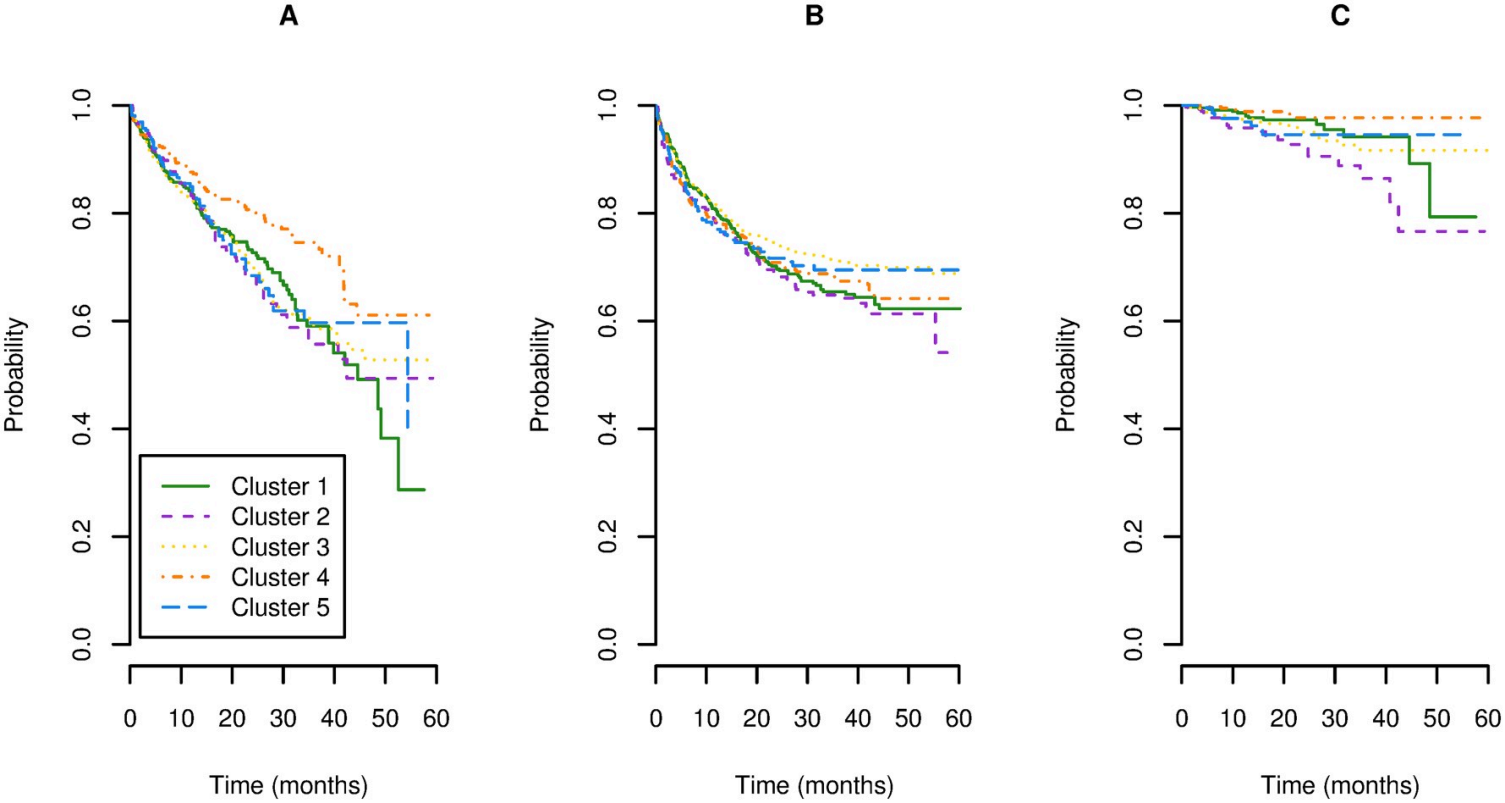
Survival Kaplan-Meier curves according to the cluster of centers. 5A. Technique failure. 5B. Peritonitis.

During the follow-up period, there were 604 technique failures, 755 peritonitis episodes occurring in 675 patients, and 81 cases of technique failure due to peritonitis. Survival curves are depicted on Fig 5.

#### Technique failure

**Fig 6:**
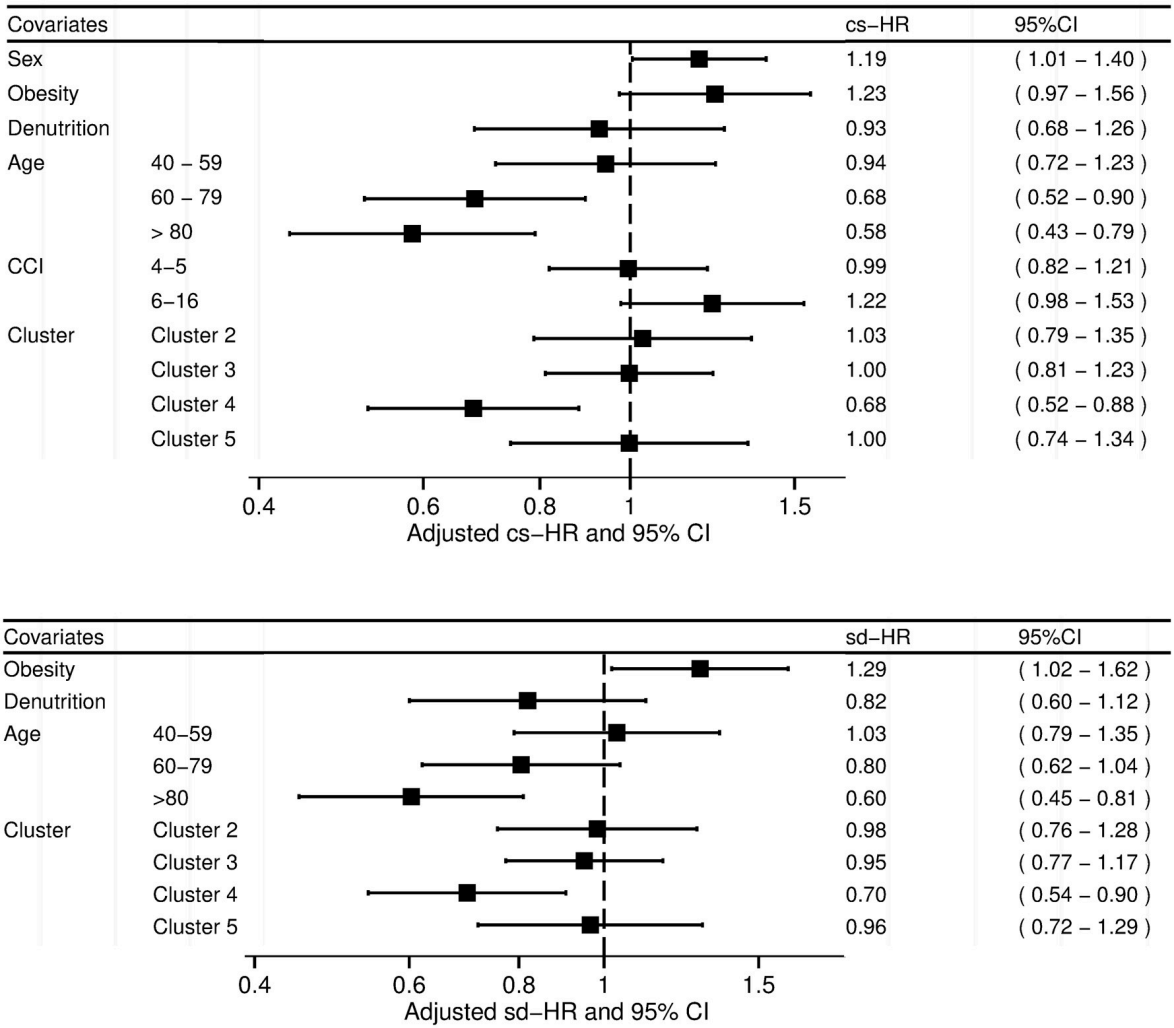
Association between covariates and technique failure. Results of the multivariate Cox model (A) and Fine and Gray model (B). The class of references are: female for the sex, 18–39 years old for the age, 2–3 for the modified Charlson comorbidity index, and cluster number 1 for the clusters of centers. CCI: modified Charlson Comorbidity Index, cs-HR: cause specific hazard-ratio, 95%CI: 95% confidence interval, sd-HR: subdistribution specific hazard-ratio.

The absolute rate of survival free of technique failure censored for transplantation and death was 87% at one year during the follow-up period. In the bivariate analysis, covariates significantly associated with the risk of technique failure were sex, obesity, malnutrition, age, Charlson comorbidity index, and belonging to cluster number 4 in the Cox model, and obesity, malnutrition, age and belonging to cluster 4 in the Fine and Gray model (Table 3). In the multivariate analysis, covariates associated with the risk of technique failure were sex and age in the Cox model. In the Fine and Gray model obesity and age were associated with the risk of technique failure (Fig 6). Being treated in a PD-unit belonging to the fourth cluster was associated with a lower risk in the Cox model (cause specific-HR = 0.68, 95%CI 0.52–0.88) and in the Fine and Gray model (sub distribution-HR = 0.70, 95%CI 0.54–0.90). Making the distinction between early or late technique failures, we found that the clusters of centers were not associated with different risks of early technique failure (S1 Table and S2 Fig). On the other side, cluster 4 was protective against the risk of late technique failure (sub distribution-HR 0.62; 95%CI 0.46–0.85) (S1 Table and S4 Fig). The results of the bivariate analysis concerning the composite outcome of technique failure and mortality are shown in S2 Table. Cluster 4 was still protective in the multivariate analysis, with the Cox model (cause specific-HR 0.80; 95%CI 0.67–0.95), and the Fine and Gray model (subdistribution-HR 0.81; 95%CI 0.68–0.96) (S4 Fig).

**Table 3:** Bivariate analysis for the three events of interest. Results of the Cox and Fine and Gray models.

	Technique failure		Peritonitis		Technique Failure due to peritonitis
Model used	Cox	Fine and Gray	Cox	Fine and Gray	Fine and Gray
	Cs-HR (95%CI)	Sd-HR (95%CI)	Cs-HR (95%CI)	Sd-HR (95%CI)	Sd-HR (95%CI)
**Covariates**					
**Sex (Male)**	1.16 (0.99–1.37)*	1.09 (0.93–1.08)	1.10 (0.95–1.28)*	1.06 (0.88–1.28)	1.75 (1.07–2.85)**
**Obesity**	1.16 (0.92–1.46)*	1.21 (0.97–1.52)*	1.59 (1.31–1.93)**	1.70 (1.33–2.17)**	0.47 (0.19–1.15)*
**Malnutrition**	0.81 (0.60–1.1)*	0.70 (0.52–0.95)**	0.95 (0.74–1.20)	1.34 (1.02–1.77)**	0.47 (0.17–1.29)*
**Age**	P < 0.001**	P < 0.001**	P = 0.03**	P < 0.001**	P = 0.22
**18–39**	Ref.	Ref.	Ref.	Ref.	Ref.
**40–59**	0.93 (0.71–1.22)	1.03 (0.79–1.34)	0.75 (0.59–0.97)**	1.02 (0.67–1.57)	2.04 (0.84–4.92)*
**60–79**	0.71 (0.55–0.91)**	0.80 (0.62–1.03)*	0.75 (0.59–0.94)*	1.65 (1.13–2.43)**	1.50 (0.63–3.54)
**> 80**	0.58 (0.44–0.78)**	0.58 (0.43–0.78)**	0.68 (0.53–0.88)**	2.21 (1.49–3.27)**	0.91 (0.35–2.37)
**Diabetes**	1.10 (0.93–1.29)	1.07 (0.91–1.27)	1.08 (0.93–1.25)	1.33 (1.10–1.61)**	0.92 (0.58–1.46)
**Nephropathy**	P = 0.29	P = 0.39	P = 0.28	P = 0.01*	P = 0.36
**Diabetic**	Ref.	Ref.	Ref.	Ref.	Ref.
**GN**	0.90 (0.66–1.21)	0.91 (0.67–1.23)	0.79 (0.59–1.05)*	0.54 (0.36–0.82)**	1.97 (0.86–4.54)*
**Unknown**	0.86 (0.64–1.17)	0.85 (0.63–1.16)	0.74 (0.55–0.99)**	0.77 (0.54–1.11)*	1.20 (0.47–3.09)
**TIN**	0.76 (0.50–1.16)	0.78 (0.51–1.19)	1.11 (0.78–1.57)	0.79 (0.49–1.29)	2.76 (1.08–7.03)**
**ADPKD**	0.82 (0.58–1.15)	0.80 (0.57–1.10)*	0.93 (0.69–1.27)	0.44 (0.27–0.73)**	1.67 (0.65–4.33)
**Urologic**	0.81 (0.40–1.66)	0.87 (0.42–1.79)	0.61 (0.29–1.30)	0.57 (0.22–1.47)	1.32 (0.17–10.28)
**Vascular**	0.64 (0.47–0.88)**	0.68 (0.50–0.94)*	0.85 (0.64–1.12)	0.91 (0.65–1.27)	0.81 (0.28–2.32)
**Other**	0.85 (0.69–1.05)*	0.81 (0.65–0.99)**	0.89 (0.73–1.09)	0.86 (0.67–1.10)	1.42 (0.72–2.81)
**Modified CCI**	P = 0.15*	P = 0.32	P = 0.93	P < 0.001**	P = 0.79
**2–3**	Ref.	Ref.	Ref.	Ref.	Ref.
**4–5**	0.88 (0.73–1.06)*	0.87 (0.73–1.05)*	1.03 (0.88–1.22)	1.51 (1.23–1.86)**	0.84 (0.50–1.40)
**5–16**	1.10 (0.90–1.36)	1.00 (0.81–1.23)	1.00 (0.83–1.22)	1.48 (1.16–1.90)**	0.97 (0.55–1.73)
**Cluster**	P = 0.005**	P = 0.04**	P = 0.17	P = 0.01	P = 0.003**
**Cluster 1**	Ref.	Ref.	Ref.	Ref.	Ref.
**Cluster 2**	1.05 (0.80–1.37)	1.03 (0.79–1.34)	1.11 (0.88–1.42)	0.95 (0.69–1.31)	2.51 (1.30–4.84)**
**Cluster 3**	1.00 (0.82–1.24)	1.00 (0.81–1.22)	0.85 (0.70–1.03)	0.78 (0.61–1.00)*	1.32 (0.72–2.41)
**Cluster 4**	0.67 (0.51–0.86)	0.70 (0.54–0.90)**	1.00 (0.81–1.24)	1.20 (0.92–1.56)*	0.46 (0.18–1.17)
**Cluster 5**	0.98 (0.74–1.32)	1.00 (0.75–1.33)	0.94 (0.72–1.23)	0.74 (0.51–1.08)*	1.36 (0.60–3.09)

Cs-HR: Cause specific hazard ratio; sd-HR: sub distribution hazard ratio; p: global p-value; GN: Glomerulonephritis; CCI: Charlson comorbidity index; PD: peritoneal dialysis;

*: p < 0.2;

**: p < 0.05

#### Peritonitis

Of the 755 first episodes of peritonitis, 348 were due to Gram-positive cocci, 188 to Gram-negative bacilli, 18 to Gram-positive bacilli, 6 to fungi, and 195 to unidentified organism. The results of the bivariate analysis are given in Table 3. In the Cox multivariate analysis, there was no significant difference between the five clusters for the peritonitis risk neither in the Cox nor in the Fine and Gray model (S5 Fig).

#### Technique failure due to peritonitis

Taking into account the competing events with a Fine and Gray model, cluster 2 was associated with a greater risk of technique failure due to peritonitis in the bivariate analysis (Table 3) as in the multivariate analysis (sub distribution-HR 2.48, 95%CI 1.29–4.78) (S6 Fig).

### Hierarchical modeling

Considering technique failure, the SD of the random effect was 0.31 in model 0. This indicates that before any adjustments, the patients treated in centers with a random effect greater than 1 SD above the mean had a relative risk of technique failure higher than 1.36 (exponential of 0.31). The variance of the random effect decreased by 5% after adjusting for patient characteristics and by 26% after adjusting for patient characteristics and center clusters. Model 1 and model 2 were significantly different from model 0 according to ANOVA (p = 3·10^−3^ and p = 2·10^−3^, respectively). These results suggest that the clusters of practices explained a fifth of the center effect. Being treated in the fourth cluster was associated with a lower risk of technique failure (cause specific-HR 0.70, 95%CI 0.50–1.00) (Table 4).

**Table 4:** Hierarchical analysis for the primary outcome: Technique failure (multivariate analysis with a mixed effect Cox model).

	Model 0 Empty model	Model 1 cs-HR (95%CI)	Model 2 cs-HR (95%CI)
Level 1 covariate			
Sex: Male	-	1.19 (1.00–1.41)*	1.19 (1.01–1.42)*
Obesity	-	1.23 (0.96–1.58)	1.25 (0.97–1.60)
Malnutrition	-	1.02 (0.74–1.40)	1.03 (0.75–1.43)
Age at PD initiation	-		
18–39		Ref.	Ref.
40–59		0.95 (0.72–1.26)	0.96 (0.73–1.28)
60–79		0.67 (0.5–0.89)*	0.68 (0.51–0.91)*
>80		0.60 (0.43–0.82)*	0.60 (0.44–0.83)*
Nephropathy			
Diabetic		Ref.	Ref.
GN		0.79 (0.55–1.15)	0.80 (0.55–1.16)
Unknown		0.84 (0.58–1.23)	0.84 (0.58–1.23)
TIN		0.69 (0.43–1.12)	0.69 (0.43–1.12)
ADPKD		0.74 (0.48–1.13)	0.74 (0.48–1.13)
Urologic		0.82 (0.38–1.79)	0.82 (0.38–1.78)
Vascular		0.73 (0.50–1.06)	0.74 (0.51–1.07)
Other		0.79 (0.59–1.06)	0.79 (0.59–1.06)
Diabetes	-	1.02 (0.78–1.33)	1.02 (0.78–1.33)
Modified CCI			
2–3	-	Ref.	Ref.
4–5		0.93 (0.75–1.16)	0.93 (0.75–1.16)
>5		1.07 (0.82–1.41)	1.06 (0.81–1.4)
Level 2 covariates	-		
Cluster			
Cluster 1	-	-	Ref.
Cluster 2			1.10 (0.72–1.68)
Cluster 3			1.07 (0.80–1.43)
Cluster 4			0.70 (0.50–1.00)*
Cluster 5			1.00 (0.67–1.50)
Random effect			
Standard error (variance)	0.31 (0.1)	0.3 (0.09)	0.27 (0.07)
Standard error of the variance of the random effect	0–0.45	0–0.45	0–0.86
p-value (ANOVA)	-	0.003*	0.002*
PCV (%)	-	5	26

PD: Peritoneal Dialysis; GN: Glomerulonephritis; TIN: Tubulo interstitial nephropathy. ADPKD: Autosomic dominant polycystic kidney disease. CCI: Charlson Comorbidity Index. ICC: Intraclass correlation coefficient. ANOVA: analysis of variance. PCV: Proportional change in variance.

*: p<0.05

### Differences of practices between the clusters

Compared with the first cluster, which was the reference, we found out that cluster 4 was protective against technique failure (cause specific-HR 0.68, 95%CI 0.52–0.88). In the centers from this cluster, there was a greater proportion of community hospitals, open surgery catheter placements, coiled catheters placed, use of local prophylactic antibiotics on exit-site, antiseptic use for dressing, and first dressing after catheter placement made between 5 and 14 days. On the other hand, there was fewer use of prophylactic antibiotic prior to catheter placement, and screening for nasal S. aureus presence. There was no difference on the covariates: use of assistance, PD modality, specialized surgeon for catheter placement (S5 Fig).

### Validation

Results of the bivariate Cox and Fine and Gray models at the patient’s level are shown in S3 Table. In the competing risk analysis, several practices were protective against the risk of technique failure at the patient’s level: being treated in a community hospital compared to being treated in an academic hospital (sub distribution -HR 1.25, 95%CI 1.02–1.54), or a private center (sub distribution -HR 1.42, 95%CI 1.09–1.87), open surgery for catheter placement compared with laparoscopy (sub distribution -HR 0.78, 95%CI 0.49–0.92), and use of local prophylactic antibiotic on exit-site (sub distribution -HR 0.65, 95%CI 0.51–0.82). This is consistent with the practices of cluster 4, found to be protective against the risk of technique failure in the hierarchical analysis. Use of assistance for PD exchange was protective at the patient’s level (sub distribution -HR 0.68, 95%CI 0.58–0.80), whereas there was no significant difference in the clusters analysis. Practices with a greater risk for technique failure were use of APD (sub distribution -HR 1.34, 95%CI 1.15–1.57), and use of antiseptic for dressing (sub distribution -HR 1.66, 95%CI 1.19–2.31). Eventually, there was no difference according to the type of PD catheter used, delay for first dressing, prophylactic antibiotic use prior to catheter placement, nasal screening for *S*. *aureus*, and surgeon experience.

## Discussion

Using an ascendant hierarchical analysis, we defined five clusters of centers with similar patterns of PD catheter related practices [10]. Our study shows that the risk of technique failure and the risk of technique failure due to peritonitis were different in these five clusters. In the hierarchical analysis, the belonging to a given cluster explained partly the center effect associated with the risk of technique failure, as the variance of the random effect decreased by 26% after adjusting for both patient characteristics and center clusters.

One should wonder why clusters of centers were used instead of studying the effect of each given practice separately. Randomized trials are mandatory to determine the best practices to propose to PD patients, but unfortunately, due to the large numbers of eligible practices, it is unlikely that evidence will be produced for each practice. Moreover, interactions may exist between practices and may have a synergic effect on the outcomes. The cluster analysis could help to identify association of practices that could influence positively the patient outcome. Furthermore, the method of clustering centers could allow implementation of quality improvement programs in group of centers, to prioritize the action at a nationwide level especially when practices are modifiable.

Cluster identification could also lead to research about the effect of pattern of practices on the patient outcome. In support of this, in the pattern of practices of cluster 4, a greater proportion of patients received local prophylactic antibiotics on exit-site, a finding in line with the ISPD guidelines, [11]. In the PanThames study, the effect of local prophylactic antibiotic use on exit-site has been assessed in twelve English PD-units in 2012. Neither mupirocin nor gentamycin reduced the peritonitis rate [Panthames]. In the cluster 4, there were more catheters placed via open surgery, and the delay for first dressing refection was between 6 to 15 days after surgery. These 2 practices have not shown their superiority in previous study to our knowledge. On the other hand, more patients in this cluster did not receive prophylactic antibiotic prior to catheter placement, whereas this practice is known to be protective against the peritonitis risk [18].

We found that cluster 4 was protective against the risk of late technique failure, whereas there was no association with the risk of early technique failure. We can hypothesize from these results that the protective effect observed in cluster 4 was mainly due to the use of local prophylactic antibiotics on exit-site, the absence of antiseptic use for dressing, and the absence of screening for nasal *S*. *aureus*, which have a sustained effect in time.

Substantial variations in the peritonitis rate are reported between countries but also within countries [19][11]. On the one hand, identifying clusters of centers with similar practices and poorer outcomes allows for a focus on centers with room for improvement because practices are modifiable. On the other hand, this method could identify units where outcomes are less favorable because of the patient characteristics.

Early peritonitis was associated with worse technique survival (HR 0.54, 95%CI 0.30–0.98) in a 10-year single-center study in Taiwan [1]. In another retrospective single-center study from China, peritonitis occurring in the first 6 months after starting PD was an independent risk factor for technique failure (HR 1.69, 95%CI 1.12–2.87) [20]. See et al. used the ANZDATA registry in a large observational study to describe the predictors and outcomes of early peritonitis. Among 3827 registered episodes of early peritonitis, 628 (16%) were followed by technique failure [4]. Other observational studies have tried to elucidate risk factors for peritonitis and poor outcomes [5] [21]. In all these studies, patient characteristics were very nearly the only variable tested, and few modifiable center-level characteristics were integrated in the analysis. However, evidence has emerged that the peritonitis risk in PD is associated with modifiable center-specific characteristics [8] [22].

Few studies have focused on PD outcomes related to center-level characteristics. In a retrospective study analyzing 9100 episodes of peritonitis from the ANZDATA registry, Htay et al. showed that center-level characteristics substantially influenced PD peritonitis outcomes. Centers with more than 29% of dialysis patients treated with PD had higher odds of cured peritonitis (odds ratio (OR) 1.21, 95%CI 1.04–1.40) and lower odds of catheter removal (OR 0.78, 95%CI 0.62–0.97), transfer to hemodialysis therapy (OR 78, 95%CI 0.62–0.97) and peritonitis relapse/recurrence (OR 0.68, 95%CI 0.48–0.98) [23]. In another retrospective multicentric study from the same team, 5813 episodes of technique failure were observed in 9642 incident patients on PD during a 10-year follow-up period. The cause of technique failure was infection in 1577 (27%) cases. Variation in the hazards of technique failure across centers was reduced by 28% after adjusting for patient-level characteristics and by an additional 53% after adjusting for center-level characteristics [24]. Guillouet et al. from our team determined in a previous study from the RDPLF data that centers characteristics accounted for 52% of the variations in early PD failure and that center size was associated with the risk of early PD failure [9].

Peritonitis risk was not different between the five clusters. However, cluster 2 was associated with a greater risk of technique failure due to peritonitis. This reflects that peritonitis outcomes are not uniform between the centers. Htay et al. found that the variation in odds of peritonitis cure across Australian centers was 66% lower after adjustment for center-level characteristics [23]. Ways of treating peritonitis could be dissimilar, or transfer to hemodialysis could be considered earlier in some centers.

Taken together, these results argue that center experience and practices are more important than patient characteristics in determining major clinical outcomes in PD, such as technique failure and peritonitis outcome.

Quality improvement programs have already proven their efficacy in PD care. The overall rate of technique failure was significantly reduced in Australia and New Zealand after a 2009 national peritonitis prevention program [25]. Poor adherence to international guidelines was outlined in different countries [10] [12]. Implementation of changes in patient care is known to be arduous. Various strategies should be used to put guidelines into practice, such as educational interactive small group meetings, use of local opinion leaders and reminders, computerized decision support, financial intervention, performance feedback, and patient-mediated intervention [26]. It would be of interest to know whether a post-peritonitis re-training protocol exists in the PD-units, as advised by the ISPD [11].

Considering every practice of a PD unit and assessing the coherence of the whole program could be a further step toward improvement. The “clusters method” that we propose here does not give a definitive answer to what the more suitable practices are, but it adds significant information by identifying centers where poor outcomes should be more prone to improve, by evaluating and changing practices.

To our knowledge, this study is the first to propose a comparison of clinical outcomes among clusters of centers with similar practices. A large population of incident PD patients was included with very good quality data [14]. Robust statistical methodologies were used, allowing for an assessment of the adjustment of patient- and center-level characteristics and competing risks.

However, these strengths should be balanced against the study’s limitations, including the retrospective design and possibility of reporting bias. The use of registry data restricts the availability of clinical variables. Participation in the registry is voluntary, thus centers participating in the RDPLF may have more involvement in PD than other centers and may have different practices. Covariates declared in the registry, like specialization of the surgeon, were not defined in a standardized way, exposing to a declaration bias. The survival models used here do not allow handling for multiple events, which is a limitation considering the outcome peritonitis.

In conclusion, clusters of centers with similar patterns of practices can be identified in France. These patterns of practices are associated with significantly different risks of technique failure. Belonging to a given cluster explained a significant part of the center effect associated with the risk of technique failure, and therefore combinations of profitable practices are suggested.

## Supporting information

S1 Table Early and late technique failure. Bivariate Cox and Fine and Gray analyses. Early technique failure is defining as occurring earlier than 3 months after the starting of PD. Late technique failure is defining as occurring later than 3 months after the starting of PD. Cs-HR: Cause specific hazard ratio; sd-HR: sub distribution hazard ratio; p: global p-value; GN: Glomerulonephritis; TIN: tubulointertitial nephritis; ADPKD: autosomic dominant polycystic disease; CCI: Charlson comorbidity index, *: p < 0.2; **: p < 0.05.
(DOCX)

S2 Table Composite outcome of technique failure and mortality bivariate Cox and Fine and Gray analyses. Cs-HR: Cause specific hazard ratio; sd-HR: sub distribution hazard ratio; GN: Glomerulonephritis; TIN: tubulointertitial nephritis; ADPKD: autosomic dominant polycystic disease; CCI: Charlson comorbidity index, *: p < 0.2; **: p < 0.05.
(DOCX)

S3 Table Validation analysis. Bivariate Cox and Fine and Gray analysis for technique failure according to practices. Hosp: hospital, CAPD: Continuous Ambulatory Peritoneal Dialysis. APD: Automated Peritoneal Dialysis. *: p-value < 0.05.
(DOCX)

S1 Fig Geometrical representation of a centre in the three-dimensional geographic space (A), and in the virtual space of practices (B). In the geographical space, the dimensions are e_X_, e_Y_, and e_Z_, corresponding to the longitude, latitude and altitude. In the space of practices, e_1_ may be the use of prophylactic antibiotic prior to catheter insertion, e_2_ the type of catheter placed in the center, and each e_i_ a given practice. In this space of n-dimension, a metric is built and distances between centers are computed, allowing to gathering the centers according to their proximity in term of practice.
(TIF)

S2 Fig Association between covariates and early technique failure (prior to 3 months after the starting of PD). Results of the multivariate Cox model (A), and Fine and Gray model (B). The classes of references are: female for the sex, 2–3 for the modified Charlson comorbidity index, and cluster number 1 for the clusters of centers. CCI: modified Charlson Comorbidity Index, cs-HR: cause specific hazard-ratio, 95%CI: 95% confidence interval, sd-HR: subdistribution specific hazard-ratio.
(PDF)

S3 Fig Association between covariates and late technique failure (later than 3 months after the starting of PD). Results of the multivariate Cox model (A), and Fine and Gray model (B). The classes of references are: diabetic nephropathy for the nephropathy, female for the sex, 18–39 years old for the age, and cluster number 1 for the clusters of centers. GN: glomerulopathy, TIN: tubulo interstitial nephropathy, ADPKD: Autosomic dominant polycystic kidney disease, cs-HR: cause specific hazard-ratio, 95%CI: 95% confidence interval, sd-HR: subdistribution specific hazard-ratio.
(PDF)

S4 Fig Association between covariates and composite outcome of technique failure and mortality. Results of the multivariate Cox model (A), and Fine and Gray model (B). The classes of references are: female for the sex, 18–39 years old for the age, diabetic nephropathy for the nephropathy, 2–3 for the modified Charlson comorbidity index, and cluster number 1 for the clusters of centers. GN: glomerulopathy, TIN: tubulo interstitial nephropathy, ADPKD: Autosomic dominant polycystic kidney disease, CCI: modified Charlson Comorbidity Index, cs-HR: cause specific hazard-ratio, 95%CI: 95% confidence interval, sd-HR: subdistribution specific hazard-ratio.
(PDF)

S5 Fig Association between covariates and peritonitis. Results of the multivariate Cox model (A) and Fine and Gray model (B). The classes of references are: female for the sex, 18–39 years old for the age, 2–3 for the CCI, and cluster number 1 for the clusters of centers. CCI: modified Charlson Comorbidity Index, cs-HR: cause specific hazard-ratio, 95%CI: 95% confidence interval, sd-HR: subdistribution specific hazard-ratio.
(PDF)

S6 Fig Association between covariates and technique failure due to peritonitis. Results of the multivariate Fine and Gray model. The classes of references are cluster number 1 for the clusters of centers. CCI: modified Charlson Comorbidity Index, cs-HR: cause specific hazard-ratio, 95%CI: 95% confidence interval, sd-HR: subdistribution specific hazard-ratio.
(PDF)

## References

[1] Hsieh YP, Wang SC, Chang CC, Wen YK, Chiu PF, Yang Y. The negative impact of early peritonitis on continuous ambulatory peritoneal dialysis patients. *Perit Dial Int* 34:627–635, 2014 10.3747/pdi.2013.00024 24497590 PMC4164407

[2] Bechade C, Guittet L, Evans D, Verger C, Ryckelynck JP, Lobbedez T. Early failure in patients starting peritoneal dialysis: a competing risks approach. *Nephrol Dial Transplant*. 29: 2127–2135, 2014 10.1093/ndt/gft055 24071660

[3] Davenport A. Peritonitis remains the major clinical complication of peritoneal dialysis: the London, UK, peritonitis audit 2002–2003. *Perit Dial Int* 29:297–302, 2009 19458302

[4] See ES, Johnson DW, Hawley CM, Pascoe EM, Darssan D, Clayton PA et al Early peritonitis and its outcome in incident peritoneal dialysis patients. *Perit Dial Int* 2018 [in press] 10.3747/pdi.2017.00029 28970365

[5] Vargas E, Blake PG, Sanabria M, Bunch A, Lopez P, Vesga J et al Early peritonitis in a large peritoneal dialysis provider system in Columbia. *Perit Dial Int* 37:30–34, 2017 10.3747/pdi.2016.00030 27605683

[6] Wu H, Huang R, Yi C, Wu J, Guo Q, Zhou Q et al Factors for early-onset peritonitis in southern chinese peritoneal dialysis patients. *Perit Dial Int* 36:640–646, 2016 10.3747/pdi.2015.00203 27147289 PMC5174871

[7] Lambie M, Davis SJ. Are peritoneal dialysis center characteristics a modifiable risk factor to improve peritoneal dialysis outcomes? *Clin J Am Soc Nephrol* 12:1032–1034, 2017 10.2215/CJN.05260517 28637864 PMC5498350

[8] Bechade C, Guillouët S, Verger C, Ficheux M, Lanot A, Lobbedez T. Center characteristics associated with risk of peritonitis in peritoneal dialysis: a hierarchical modelling approach based on the data of the French Language Peritoneal Dialysis Registry (RDPLF). *Nephrol Dial Transplant* 32:1018–1023, 2017 10.1093/ndt/gfx051 28472525

[9] Guillouet S, Veniez G, Verger C, Bechade C, Ficheux M, Uteza J et al Estimation of the center effect on early peritoneal dialysis failure: a multilevel modelling approach. *Perit Dial Int* 36: 519–525, 2016 10.3747/pdi.2015.00245 27044794 PMC5033627

[10] Lanot A, Bechade C, Verger C, Fabre E, Vernier I, Lobbedez T et al Clusters of practice in peritoneal dialysis in France: Data from the catheter section of the RDPLF. *Perit Dial Int* 38: 89–97, 2018 10.3747/pdi.2017.00135 29162681

[11] Li PK, Szeto CC, Piraino B, Arteaga J, Fan S, Figueiredo AE. ISPD peritonitis recommendations: 2016 update on prevention and treatment. *Perit Dial Int* 36:481–508, 2016 10.3747/pdi.2016.00078 27282851 PMC5033625

[12] Campbell DJ, Brown FG, Craig JC, Gallagher MP, Johnson DW, Kirkland GS et al Assessment of current practice and barriers to antimicrobial prophylaxis in peritoneal dialysis patient. *Nephrol Dial Transplant* 31:619–627, 2015 10.1093/ndt/gfv115 25906780

[13] Cho Y, Htay H, Johnson DW. Centre effects and peritoneal dialysis-related peritonitis. *Nephrol Dial Transplant* 32(6):907–915, 2017 28505351 10.1093/ndt/gfx054

[14] Verger C, Ryckelynck JP, Duman M, Veniez G, Lobbedez T, Boulanger E et al French peritoneal dialysis registry (RDPLF): outline and main results. *Kidney Int*. *suppl*. 103: S12–20, 2006 10.1038/sj.ki.5001911 17080102

[15] Ward JH. Hierarchical grouping to optimize an objective function. *J Am Stat Assoc* 58:236–44, 1963

[16] Lau B, Cole SR, Gange SJ. Competing risk regression models for epidemiologic data. Am J Epidemiol 2009;170:244–256 10.1093/aje/kwp107 19494242 PMC2732996

[17] Von Elm E, Altman DG, Egger M, Pocock SJ, Gøtzsche PC, Vandenbroucke JP. The Strengthening the Reporting of Observational Studies in Epidemiology (STROBE) statement: guidelines for reporting observational studies. *J Clin Epidemiol* 61:344–9, 2008 10.1016/j.jclinepi.2007.11.008 18313558

[18] Strippoli GF, Tong A, Johnson D, Schena FP, Craig JC. Antimicrobial agents to prevent peritonitis in peritoneal dialysis: a systematic review of randomized controlled trials. Am J Kidney Dis 44: 591–603, 2004 15384009

[19] Piraino B, Bernardini J, Brown E, Figueiredo A, Johnson DW, Lye WC et al ISPD Position Statement on Reducing the Risks of Peritoneal Dialysis–Related Infections. *Perit Dial Int*. 31:614–30, 2011 10.3747/pdi.2011.00057 21880990

[20] Feng S, Wang Y, Qiu B, Wang Z, Jiang L, Zhan Z et al Impact of early-onset peritonitis on mortality and technique survival in peritoneal dialysis patients. *Springerplus* 5:1676, 2016 10.1186/s40064-016-3369-9 27733978 PMC5040655

[21] Pulliam J, Li N-C, Maddux F, Hakim R, Finkelstein FO, Lacson E. First-year outcomes of incident peritoneal dialysis patients in the United States. *Am J Kidney Dis* 64:761–9, 2014 10.1053/j.ajkd.2014.04.025 24927898

[22] Nadeau-Fredette A-C, Johnson DW, Hawley CM, Pascoe EM, Cho Y, Clayton PA et al Center specific factors associated with peritonitis risk—a multi-center registry analysis. *Perit Dial Int*. 36:509–518, 2016 10.3747/pdi.2015.00146 26764341 PMC5033626

[23] Htay H, Cho Y, Pascoe EM, Darssan D, Nadeau-Fredette AC, Hawley C et al Center effects and peritoneal dialysis peritonitis outcomes: analysis of a national registry. *Am J Kidney Dis* 2018;71:814–821 10.1053/j.ajkd.2017.10.017 29289475

[24] Htay H, Cho Y, Pascoe EM, Darssan D, Nadeau-Fredette AC, Hawley C et al Multicenter registry analysis of center characteristics associated with technique failure in patients on incident peritoneal dialysis. *Clin J Am Soc Nephrol* 12:1090–1099, 2017 10.2215/CJN.12321216 28637862 PMC5498362

[25] Mudge DW, Boudville N, Brown F, Clayton P, Duddington M, Holt S et al Peritoneal dialysis practice in Australia and New Zealand: a call to sustain the action. *Nephrology (Carlton)* 21:535–546, 2016 26807739 10.1111/nep.12731

[26] Grol R, Grimshaw J. From best evidence to best practice: effective implementation of change in patients’ care. *Lancet* 302:1225–1230, 2003 10.1016/S0140-6736(03)14546-1 14568747

