# Supplementary material for: Patterns of peritoneal dialysis catheter practices and technique failure in peritoneal dialysis: A nationwide cohort study
Source: PLoS One. 2019 Jun 20;14(6):e0218677. doi: 10.1371/journal.pone.0218677 (PMC6586404; doi:10.1371/journal.pone.0218677)
Supplement: S1 Table — Early technique failure is defining as occurring earlier than 3 months after the starting of PD. Late technique failure is defining as occurring later than 3 months after the starting of PD. Cs-HR: Cause specific hazard ratio; sd-HR: sub distribution hazard ratio; p: global p-value; GN: Glomerulonephritis; TIN: tubulointertitial nephritis; ADPKD: autosomic dominant polycystic disease; CCI: Charlson comorbidity index, *: p < 0.2; **: p < 0.05. (DOCX) [file pone.0218677.s001.docx]

**Table S1. Early and late technique failure. Bivariate Cox and Fine and Gray analyses.**

|  | **Early technique failure** | | **Late technique failure** | |
| --- | --- | --- | --- | --- |
| **Model used** | **Cox** | **Fine and Gray** | **Cox** | **Fine and Gray** |
|  | **Cs-HR (95%CI)** | **Sd-HR (95%CI)** | **Cs-HR (95%CI)** | **Sd-HR (95%CI)** |
| **Covariates** |  |  |  |  |
| **Sex (Male)** | 0.74 (0.54-1.02) | 0.75 (0.55-1.03) | 1.36 (1.12-1.65)** | 1.26 (1.04-1.53)** |
| **Obesity** | 1.08 (0.68-1.74) | 1.15 (0.72-1.81) | 1.19 (0.91-1.54)* | 1.22 (0.94-1.58)* |
| **Malnutrition** | 1.14 (0.69-1.88) | 1.10 (0.67-1.83) | 0.69 (0.48-1.01)* | 0.58 (0.40-0.84)** |
| **Age** | P=0.36 | P=0.44 | P<0.001 | P < 0.001 |
| **18-39** | Ref. | Ref. | Ref. | Ref. |
| **40-59** | 0.80 (0.44-1.44) | 0.81 (0.45-1.45) | 0.96 (0.71-1.30) | 1.11 (0.82-1.50) |
| **60-79** | 0.85 (0.49-1.46) | 0.86 (0.50-1.47) | 0.66 (0.50-0.89)** | 0.79 (0.59-1.06) |
| **> 80** | 1.14 (0.65-1.98) | 1.11 (0.64-1.93) | 0.43 (0.30-0.61)** | 0.43 (0.30-0.61)** |
| **Diabetes** | 1.11 (0.80-1.54) | 1.08 (0.78-1.51) | 1.09 (0.90-1.33) | 1.06 (0.88-1.29) |
| **Nephropathy** | P=0.64 | P=0.59 | P=0.13 | P=0.22 |
| **Diabetic** | Ref. | Ref. | Ref. | Ref. |
| **GN** | 1.12 (0.62-2.02) | 1.12 (0.62- 2.03) | 0.83 (0.59-1.18) | 0.84 (0.59-1.20) |
| **Unknown** | 1.14 (0.64-2.04) | 1.20 (0.68-2.12) | 0.78 (0.55-1.12)* | 0.74 (0.52-1.07) |
| **TIN** | 1.13 (0.52-2.46) | 1.14 (0.53-2.48) | 0.66 (0.39-1.10)* | 0.67 (0.40-1.11) |
| **ADPKD** | 0.45 (0.17-1.15) | 0.45 (0.17-1.16) | 0.92 (0.63-1.33) | 0.92 (0.64-1.34) |
| **Urologic** | 1.69 (0.52-5.52) | 1.64 (0.51-5.33) | 0.61 (0.25-1.51) | 0.66 (0.27-1.59) |
| **Vascular** | 0.97 (0.53-1.77) | 0.96 (0.53-1.76) | 0.56 (0.38-0.81)** | 0.61 (0.42-0.89) |
| **Other** | 0.91 (0.59-1.41) | 0.90 (0.58-1.39) | 0.83 (0.65-1.06)* | 0.79 (0.62-1.00) |
| **Modified CCI** | P=0.18 | P=0.23 | P=0.51 | P=0.64 |
| **2 - 3**  **4 - 5**  **5 - 16** | Ref.  0.79 (0.54-1.15)  1.20 (0.81-1.78) | Ref.  0.80 (0.55-1.17)  1.18 (0.80-1.76) | Ref.  0.91 (0.74-1.13)  1.07 (0.83-1.36) | Ref.  0.91 (0.74-1.12)  0.93 (0.72-1.18) |
| **Cluster** | P=0.56 | P=0.50 | P<0.001 | P<0.001 |
| **Cluster 1** | Ref. | Ref. | Ref. | Ref. |
| **Cluster 2** | 0.72 (0.38-1.35) | 0.72 (0.38-1.34) | 1.14 (0.85-1.54) | 1.15 (0.86-1.55) |
| **Cluster 3** | 1.07 (0.72-1.60) | 1.10 (0.74-1.63) | 0.99 (0.78-1.25) | 0.96 (0.76-1.22) |
| **Cluster 4** | 0.97 (0.60-1.55) | 0.97 (0.60-1.55) | 0.58 (0.42-0.79)** | 0.62 (0.46-0.85)** |
| **Cluster 5** | 0.69 (0.35-1.34) | 0.69 (0.36-1.35) | 1.08 (0.78-1.49) | 1.12 (0.81-1.55) |

Early technique failure is defining as occurring earlier than 3 months after the starting of PD. Late technique failure is defining as occurring later than 3 months after the starting of PD. Cs-HR: Cause specific hazard ratio; sd-HR: sub distribution hazard ratio; p: global p-value; GN: Glomerulonephritis; TIN: tubulointertitial nephritis; ADPKD: autosomic dominant polycystic disease; CCI: Charlson comorbidity index, *: p < 0.2; **: p < 0.05
