# Supplementary material for: Patterns of peritoneal dialysis catheter practices and technique failure in peritoneal dialysis: A nationwide cohort study
Source: PLoS One. 2019 Jun 20;14(6):e0218677. doi: 10.1371/journal.pone.0218677 (PMC6586404; doi:10.1371/journal.pone.0218677)
Supplement: S2 Table — Cs-HR: Cause specific hazard ratio; sd-HR: sub distribution hazard ratio; GN: Glomerulonephritis; TIN: tubulointertitial nephritis; ADPKD: autosomic dominant polycystic disease; CCI: Charlson comorbidity index, *: p < 0.2; **: p < 0.05. (DOCX) [file pone.0218677.s002.docx]

**Table S2. Composite outcome of technique failure and mortality Bivariate Cox and Fine and Gray analyses.**

|  | **Technique failure and mortality** | |
| --- | --- | --- |
| **Model used** | **Cox** | **Fine and Gray** |
|  | **Cs-HR (95%CI)** | **Sd-HR (95%CI)** |
| **Covariates** |  |  |
| **Sex (Male)** | 1.19 (1.06-1.34) | 1.14 (1.02-1.29) |
| **Obesity** | 1.19 (1.01-1.40) | 1.31 (1.12-1.53) |
| **Malnutrition** | 1.65 (1.40-1.94) | 1.82 (1.56-2.13) |
| **Age** |  |  |
| **18-39** | Ref. | Ref. |
| **40-59** | 0.97 (0.75-1.25) | 1.11 (0.86-1.44) |
| **60-79** | 1.26 (1.00-1.59) | 1.75 (1.38-2.22) |
| **> 80** | 1.84 (1.46-2.33) | 2.74 (2.16-3.49) |
| **Diabetes** | 1.44 (1.29-1.62) | 1.66 (1.48-1.86) |
| **Nephropathy** |  |  |
| **Diabetic** | Ref. | Ref. |
| **GN** | 0.63 (0.49-0.80) | 0.53 (0.41-0.67) |
| **Unknown** | 0.86 (0.69-1.06) | 0.79 (0.63-0.98) |
| **TIN** | 0.54 (0.38-0.76) | 0.45 (0.32-0.63) |
| **ADPKD** | 0.48 (0.36-0.65) | 0.37 (0.28-0.49) |
| **Urologic** | 0.72 (0.43-1.21) | 0.72 (0.40-1.28) |
| **Vascular** | 0.83 (0.68-1.02) | 0.87 (0.72-1.05) |
| **Other** | 0.86 (0.74-1.00) | 0.77 (0.67-0.89) |
| **Modified CCI** |  |  |
| **2 - 3** | Ref. | Ref. |
| **4 – 5** | 1.48 (1.29-1.69) | 1.76 (1.54-2.01) |
| **5 - 16** | 2.06 (1.79-2.38) | 2.53 (2.19-2.91) |
| **Cluster** |  |  |
| **Cluster 1** | Ref. | Ref. |
| **Cluster 2** | 1.11 (0.92-1.34) | 1.09 (0.91-1.30) |
| **Cluster 3** | 0.90 (0.77-1.04) | 0.83 (0.71-0.96) |
| **Cluster 4** | 0.81 (0.68-0.97) | 0.84 (0.71-0.96) |
| **Cluster 5** | 0.77 (0.61-0.96) | 0.69 (0.55-0.86) |

Cs-HR: Cause specific hazard ratio; sd-HR: sub distribution hazard ratio; GN: Glomerulonephritis; TIN: tubulointertitial nephritis; ADPKD: autosomic dominant polycystic disease; CCI: Charlson comorbidity index, *: p < 0.2; **: p < 0.05
