# Supplementary material for: Patterns of peritoneal dialysis catheter practices and technique failure in peritoneal dialysis: A nationwide cohort study
Source: PLoS One. 2019 Jun 20;14(6):e0218677. doi: 10.1371/journal.pone.0218677 (PMC6586404; doi:10.1371/journal.pone.0218677)
Supplement: S3 Table — Hosp: hospital, CAPD: Continuous Ambulatory Peritoneal Dialysis. APD: Automated Peritoneal Dialysis. *: p-value < 0.05. (DOCX) [file pone.0218677.s003.docx]

**Table S3. Validation analysis. Bivariate Cox and Fine and Gray analysis for technique failure according to practices.**

| **Technique failure** |  |  |  |
| --- | --- | --- | --- |
| Covariate |  | Cox: | Fine & Gray: |
|  |  | cs-HR [IC95] | sd-HR[IC95] |
| Administrative Structure | Community hosp. | Ref. | Ref. |
|  | Non profit | 1.14 [0.93-1.39] | 0.90 [0.91-1.35] |
|  | Academic hosp. | 1.33 [1.08-1.64]* | 1.25 [1.02-1.54]* |
|  | Private | 1.33 [1.00-1.76]* | 1.42 [1.09-1.87]* |
| Surgical technique | Laparoscopy | Ref. | Ref. |
|  | Open surgery | 0.75 [0.63-0.89]* | 0.78 [0.49-0.92]* |
|  | Trocart | 1.10 [0.41-2.96] | 1.21 [0.49-3.03] |
| Type of catheter | Coiled | Ref. | Ref. |
|  | Straight | 1.1 [0.93-1.30] | 1.11 [0.94-1.3] |
| Local prophylactic antibiotics on exit-site | No antibiotic | Ref. | Ref. |
|  | Antibiotic | 0.68 [0.53-0.86]* | 0.65 [0.51-0.82]* |
| Antiseptic used for dressing | No antiseptic | Ref. | Ref. |
|  | Antiseptic | 1.72 [1.23-2.4]* | 1.66 [1.19-2.31]* |
| Delay for first dressing | 0 to 5 days | Ref. | Ref. |
|  | 6 to 15 days | 0.84 [0.71-0.99]* | 0.86 [0.73-1.01] |
|  | After day 16 | 1.84 [0.59-5.75] | 1.82 [0.64-5.2] |
| Prophylactic antibiotic for catheter placement | No antibiotic | Ref. | Ref. |
|  | Antibiotic | 1.20 [1.02-1.41]* | 1.14 [0.97-1.33] |
| Nasal *S.aureus* screening | No screening | Ref. | Ref. |
|  | Screening | 0.99 [0.84-1.17] | 1.00 [0.85-1.18] |
| Assistance | Autonomous | Ref. | Ref. |
|  | Assisted | 0.74 [0.63-0.87]* | 0.68 [0.58-0.80]* |
| PD modality | CAPD | Ref. | Ref. |
|  | APD | 1.25 [1.07-1.47]* | 1.34 [1.15-1.57]* |
| Surgeon | Not specialized | Ref. | Ref. |
|  | Specialized | 0.79 [0.61-1.04] | 0.79 [0.61-1.03] |

Hosp: hospital, CAPD: Continuous Ambulatory Peritoneal Dialysis. APD: Automated Peritoneal Dialysis. *: p-value < 0.05
