# Supplementary figures and images for: Patterns of peritoneal dialysis catheter practices and technique failure in peritoneal dialysis: A nationwide cohort study
Source: PLoS One. 2019 Jun 20;14(6):e0218677. doi: 10.1371/journal.pone.0218677 (PMC6586404; doi:10.1371/journal.pone.0218677)

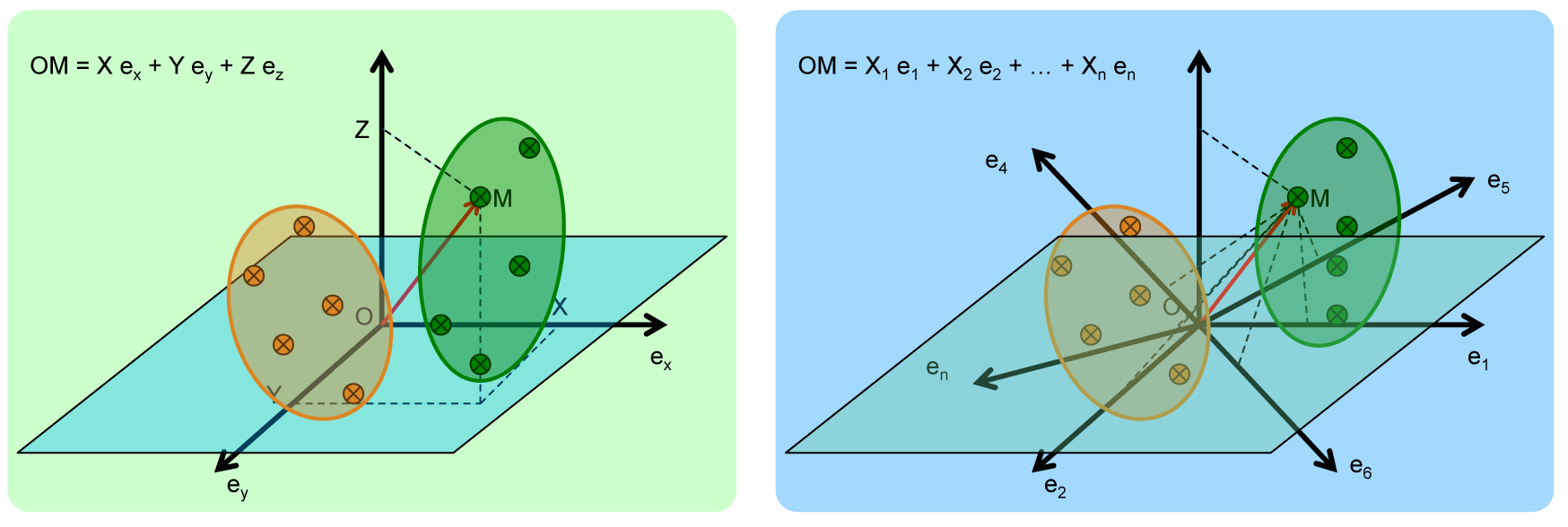

Supplement: S1 Fig — In the geographical space, the dimensions are eX, eY, and eZ, corresponding to the longitude, latitude and altitude. In the space of practices, e1 may be the use of prophylactic antibiotic prior to catheter insertion, e2 the type of catheter placed in the center, and each ei a given practice. In this space of n-dimension, a metric is built and distances between centers are computed, allowing to gathering the centers according to their proximity in term of practice. (TIF) [file pone.0218677.s004.tif]

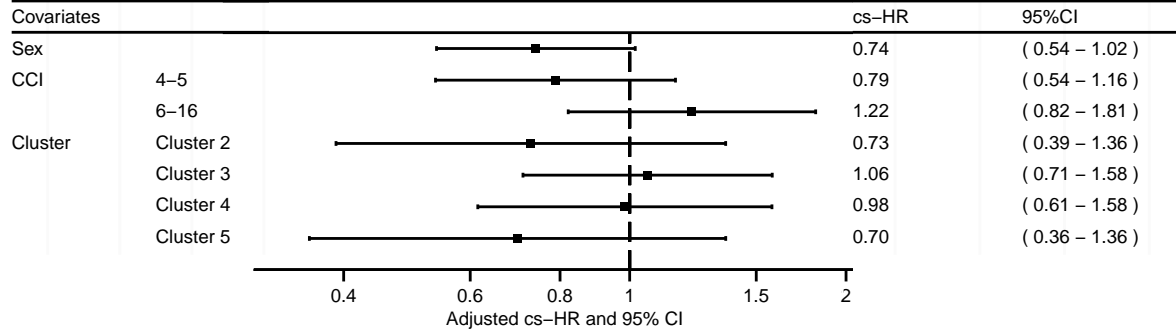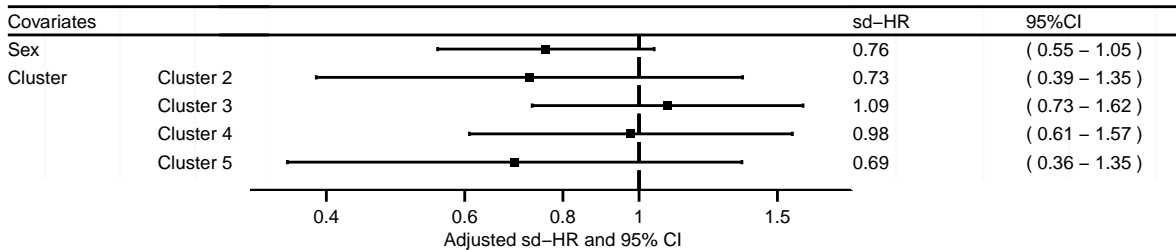

Supplement: S2 Fig — The classes of references are: female for the sex, 2–3 for the modified Charlson comorbidity index, and cluster number 1 for the clusters of centers. CCI: modified Charlson Comorbidity Index, cs-HR: cause specific hazard-ratio, 95%CI: 95% confidence interval, sd-HR: subdistribution specific hazard-ratio. (PDF) [file pone.0218677.s005.pdf]

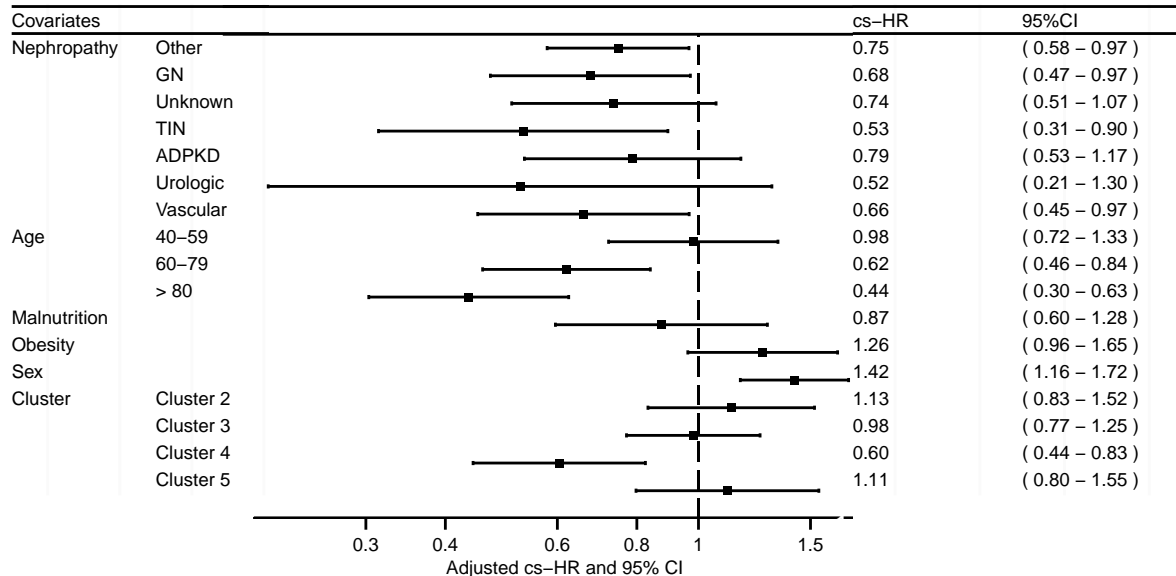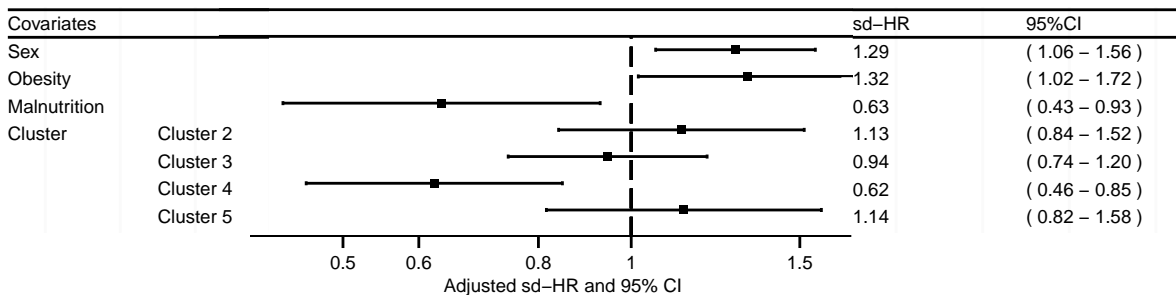

Supplement: S3 Fig — The classes of references are: diabetic nephropathy for the nephropathy, female for the sex, 18–39 years old for the age, and cluster number 1 for the clusters of centers. GN: glomerulopathy, TIN: tubulo interstitial nephropathy, ADPKD: Autosomic dominant polycystic kidney disease, cs-HR: cause specific hazard-ratio, 95%CI: 95% confidence interval, sd-HR: subdistribution specific hazard-ratio. (PDF) [file pone.0218677.s006.pdf]

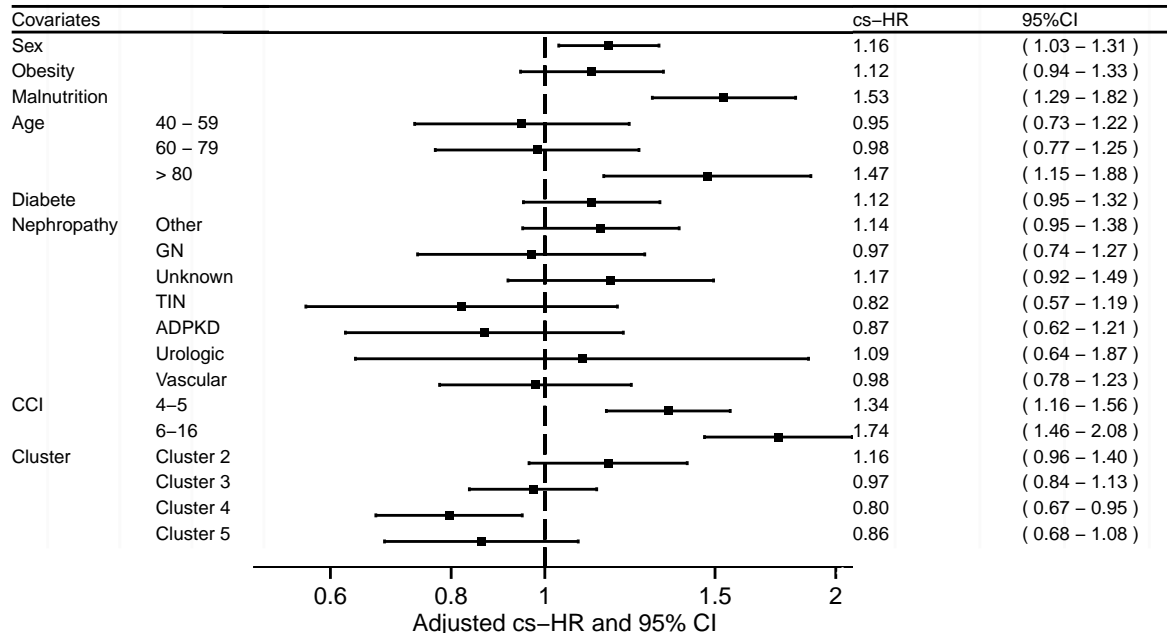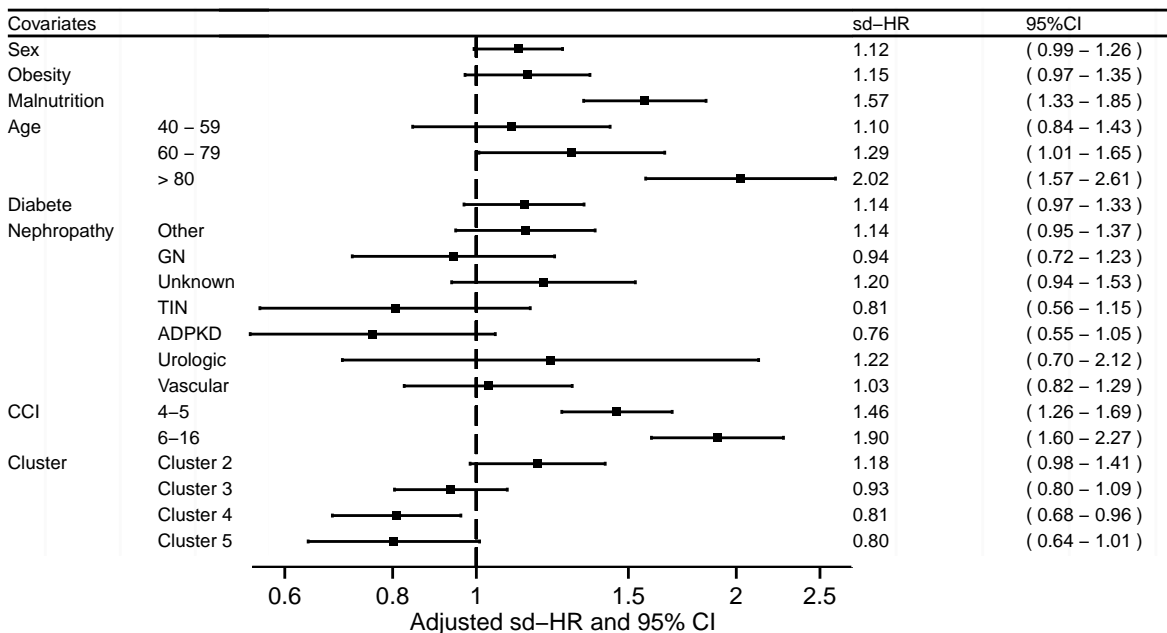

Supplement: S4 Fig — The classes of references are: female for the sex, 18–39 years old for the age, diabetic nephropathy for the nephropathy, 2–3 for the modified Charlson comorbidity index, and cluster number 1 for the clusters of centers. GN: glomerulopathy, TIN: tubulo interstitial nephropathy, ADPKD: Autosomic dominant polycystic kidney disease, CCI: modified Charlson Comorbidity Index, cs-HR: cause specific hazard-ratio, 95%CI: 95% confidence interval, sd-HR: subdistribution specific hazard-ratio. (PDF) [file pone.0218677.s007.pdf]

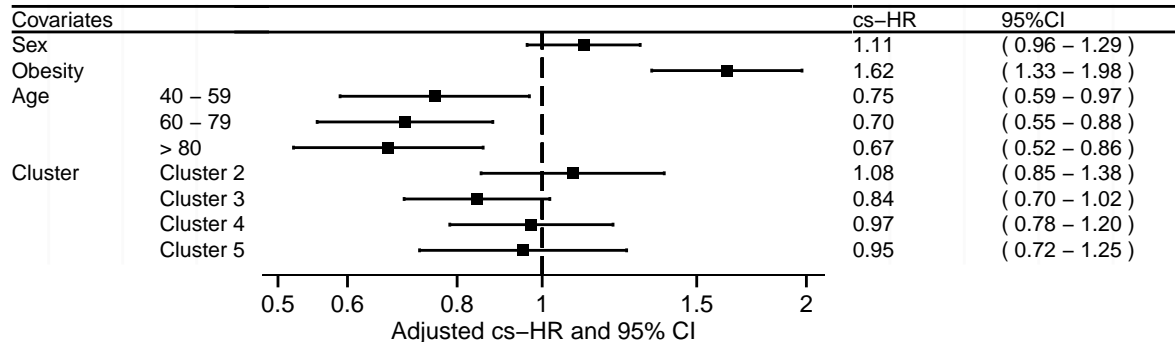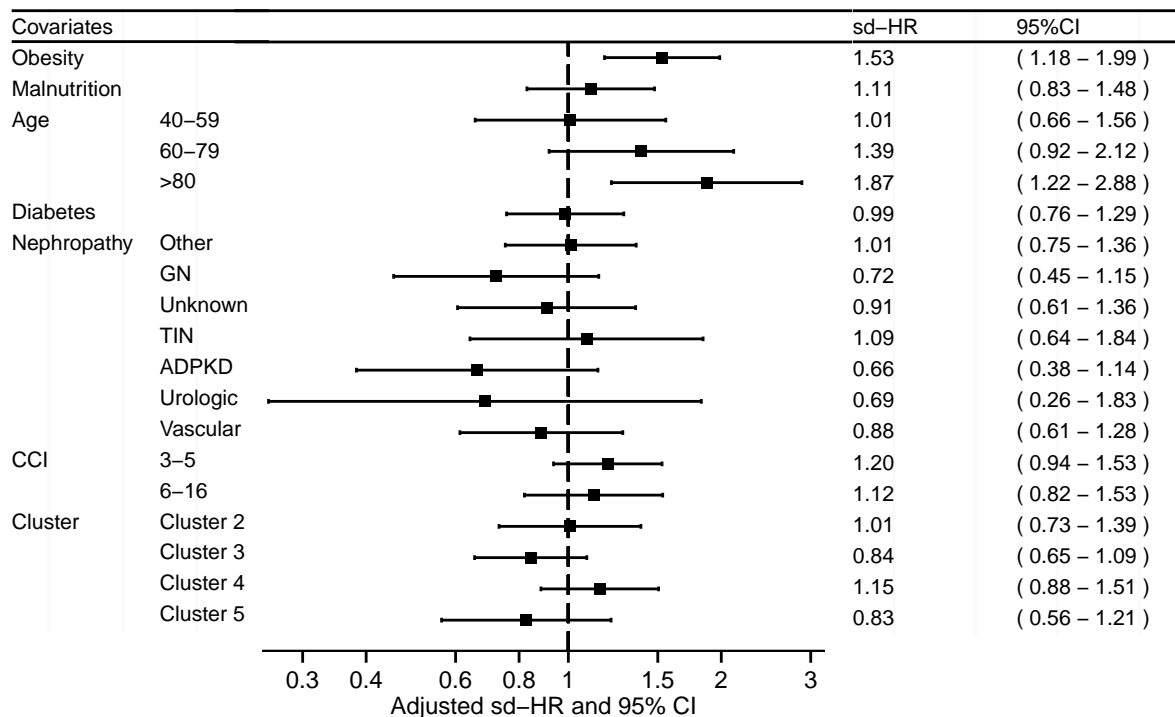

Supplement: S5 Fig — The classes of references are: female for the sex, 18–39 years old for the age, 2–3 for the CCI, and cluster number 1 for the clusters of centers. CCI: modified Charlson Comorbidity Index, cs-HR: cause specific hazard-ratio, 95%CI: 95% confidence interval, sd-HR: subdistribution specific hazard-ratio. (PDF) [file pone.0218677.s008.pdf]

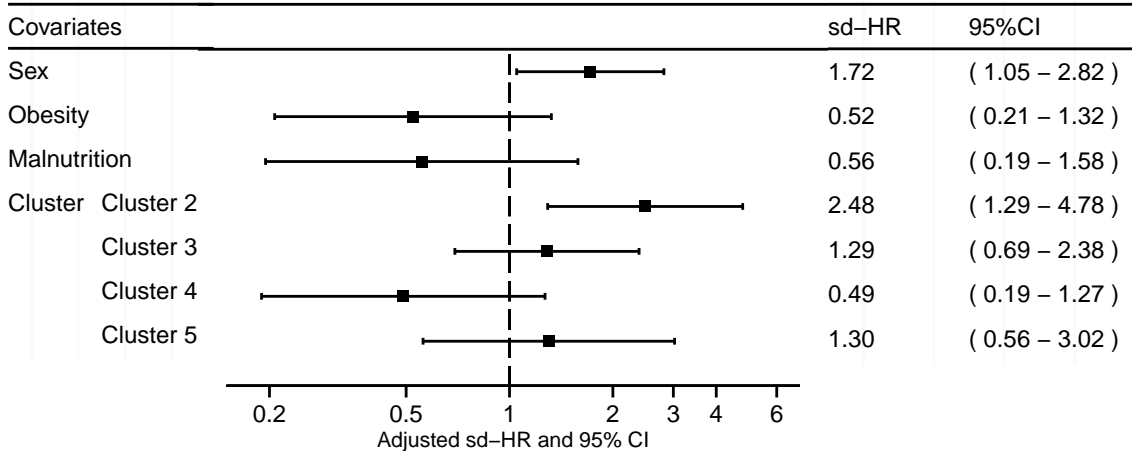

Supplement: S6 Fig — The classes of references are cluster number 1 for the clusters of centers. CCI: modified Charlson Comorbidity Index, cs-HR: cause specific hazard-ratio, 95%CI: 95% confidence interval, sd-HR: subdistribution specific hazard-ratio. (PDF) [file pone.0218677.s009.pdf]
